# Climatology and Mineral Waters of the United States

**Published:** 1886-04

**Authors:** 


Climatology and Mineral Waters of the United States. *By* A. N. Bell, a. m., m. d. *pp. 386. New York:* William Wood & Co.

This is the October number of *Wood's Library* for 1885, and is an acceptable addition to the list already published.

A work of this nature is necessarily a compilation, and the author has shown good judgment in selecting from weather reports, detached analyses, society proceedings and other sources the valuable tables and explanations here given.

